# Twitter Misinformation Discourses About Vaping: Systematic Content Analysis

**DOI:** 10.2196/49416

**Published:** 2023-11-10

**Authors:** Ahmed Al-Rawi, Breanna Blackwell, Kiana Zemenchik, Kelley Lee

**Affiliations:** 1 Simon Fraser University Burnaby, BC Canada

**Keywords:** vaping, e-cigarette, smoking, misinformation, fact checking, social media, Twitter, nicotine, content analysis, fact-checking, disinformation, weaponized, health risk, risk, health education, education, communication, electronic nicotine delivery systems, ENDS

## Abstract

**Background:**

While there has been substantial analysis of social media content deemed to spread misinformation about electronic nicotine delivery systems use, the strategic use of misinformation accusations to undermine opposing views has received limited attention.

**Objective:**

This study aims to fill this gap by analyzing how social media users discuss the topic of misinformation related to electronic nicotine delivery systems, notably vaping products. Additionally, this study identifies and analyzes the actors commonly blamed for spreading such misinformation and how these claims support both the provaping and antivaping narratives.

**Methods:**

Using Twitter’s (subsequently rebranded as X) academic application programming interface, we collected tweets referencing #vape and #vaping and keywords associated with fake news and misinformation. This study uses systematic content analysis to analyze the tweets and identify common themes and actors who discuss or possibly spread misinformation.

**Results:**

This study found that provape users dominate the platform regarding discussions about misinformation about vaping, with provaping tweets being more frequent and having higher overall user engagement. The most common narrative for provape tweets surrounds the conversation of vaping being perceived as safe. On the other hand, the most common topic from the antivape narrative is that vaping is indeed harmful. This study also points to a general distrust in authority figures, with news outlets, public health authorities, and political actors regularly accused of spreading misinformation, with both placing blame. However, specific actors differ depending on their positionalities. The vast number of accusations from provaping advocates is found to shape what is considered misinformation and works to silence other narratives. Additionally, allegations against reliable and proven sources, such as public health authorities, work to discredit assessments about the health impacts, which is detrimental to public health overall for both provaping and antivaping advocates.

**Conclusions:**

We conclude that the spread of misinformation and the accusations of misinformation dissemination using terms such as “fact check,” “misinformation,” “fake news,” and “disinformation” have become weaponized and co-opted by provaping actors to delegitimize criticisms about vaping and to increase confusion about the potential health risks. The study discusses the mixed types of impact of vaping on public health for both smokers and nonsmokers. Additionally, we discuss the implications for effective health education and communication about vaping and how misinformation claims can affect evidence-based discourse on Twitter as well as informed vaping decisions.

## Introduction

Electronic nicotine delivery systems (ENDS), also known as e-cigarettes, were introduced as a means of harm reduction or cessation tool for cigarette smokers. However, e-cigarettes and especially vaping products have also become highly popular among those who previously did not use nicotine products including adolescents and youth [1,2]. In the United States, the percentage of middle and high school students who have ever used ENDS rose from 10% in 2011 to more than 27% in 2019 [1,3,4]. Among American adolescents who currently use ENDS, 38.9% report using their devices at least 20 days per month [1]. In the United Kingdom, 32.7% of adolescents have used an ENDS device, with 18% currently using it. In Australia, the National Drug Society Household survey found that 11.3% of Australians who are 14 years of age and older had tried vaping [5]. By 2015, e-cigarettes became the most used tobacco product in the United States [4,6], with 9 million adults and youths reporting choosing them over other forms of tobacco use [6].

This rapid rise in ENDS use has raised public health concerns for several reasons. Vaping products have been found to contain nicotine, organic compounds, and other components, which have been shown to cause numerous health ailments, such as respiratory and cardiovascular problems and possibly cancer, and have been associated with seizures and acute pulmonary injuries [2-4]. Their use by people who were previously nonsmokers, notably adolescents and youth, represents an increase rather than a decrease in health harms [2-5,7,8]. The practice of dual use by existing smokers (with ENDS use in smoke-free environments) raises concerns about their true effectiveness in encouraging cessation. The high levels of nicotine in some products have prompted concerns about the harms of higher dosing and increased addiction. Evidence suggests that nicotine-containing e-cigarettes can negatively impact brain function and increase one’s susceptibility to substance addiction [3]. For this reason, nicotine-containing e-cigarettes are prohibited in Australia, and liquid nicotine is categorized as a level 7 dangerous poison [5]. Findings show that the use of e-cigarettes in youth has been associated with the subsequent use of combustibles due to nicotine addiction [3,4]. There is growing evidence of severe health consequences associated with even short-term use of vaping devices, regardless of the inclusion of nicotine [5,9]. Finally, there are concerns about the health risks from secondhand exposure to e-cigarette vapors. These concerns have prompted substantial debate between advocates and opponents of ENDS [10].

Evidence suggests substantial misinformation about e-cigarettes on social media arising from both sides of the debate. Antivaping misinformation on social media includes claims that all e-cigarette vaping products contain nicotine or exaggerations of risks [11,12]. For example, a 2021 study found that smokers were more likely to engage with tweets that suggested vaping was as harmful or more harmful than traditional cigarettes and those that implied vaping was completely safe [11].

Provaping misinformation includes claims that there are little or no health harms from using ENDS and that vaping is not addictive [11,12]. There are claims that using e-cigarettes results in a high success rate of quitting smoking and that it generates economic and environmental benefits. Other misinformation identified claims nicotine is as addictive as caffeine along with misrepresentation of e-cigarette regulations [12]. Because of the perceived effectiveness of ENDS to aid in smoking cessation, many pro-ENDS advocates claim that vaping saves lives. Allem et al [7], for example, warned that social bots may also perpetuate this narrative through misleading web-based discourse. Social bots are prominent on Twitter, where they produce large numbers of tweets, saturate certain areas of social discourse, and are often linked to the spread of misinformation [13,14]. Overall, 1 study [12] found that over 41% of tweets that shared information about vaping contained at least 1 piece of possible misinformation [12]. The result has been a lack of accurate understanding of the health risks and benefits. For example, 1 study finds that 63% of youths who use e-cigarettes are unaware that they contain nicotine, some believing that the synthetic nicotine found in ENDS does not have addictive properties and is harmless [12].

While there has been substantial analysis of social media content deemed to spread misinformation about ENDS use, the strategic use of misinformation accusations to undermine opposing views in debates about vaping has received limited attention to date. In other domains of public discourse, misinformation and disinformation accusations have been increasingly used for populist political purposes. As we observe in a study, “these accusations might have critical consequences for the public perception of authoritative information sources such as media and science, possibly undermining their role in providing citizens with the information they need” [15]. To understand the extent to which misinformation accusations shape the public discourse on social media related to vaping, we conduct a content analysis of data collected from Twitter. We investigate the sources of these accusations and the basis upon which claims of misinformation are made by analyzing tweets that contain the hashtags #vape and #vaping as well as terms such as “misinformation,” “disinformation,” and others related to fake news. Using emergent coding, we designed a codebook to identify reoccurring major topics and misinformation disseminators. Then, we coded the tweets’ positionality, topics, and actors who spread misinformation to gain insight into the web-based discourse surrounding misinformation about vaping on Twitter. We present new findings about how misinformation has become co-opted and weaponized to advance particular positions on vaping. We discuss the implications for the erosion of public understanding about the risks and benefits of ENDS and potential strategies for protecting evidence-informed policy discourse. This study seeks to answer the following research question: in relation to misinformation discourse about vaping on Twitter, what are the main topics identified and who are the major actors accused of disseminating misinformation?

## Methods

### Overview

Using Twitter’s academic application programming interface (API), we collected tweets referencing #vape and #vaping that were posted between August 1, 2006 and August 12, 2022. In total, 6,622,940 tweets were collected that were posted by 572,412 unique users. These were all the available tweets that were on Twitter by the time the search was conducted using the 2 previously mentioned hashtags, rather than the general terms with the pound sign omitted. We chose Twitter because it used to be one of the few social media platforms that allowed full historical API access; however, this academic API access was recently revoked for all researchers after Elon Musk purchased the platform. The start date of August 1, 2006, was chosen because it is close to the beginning of Twitter’s launch. The end date of August 12, 2022, is when the API search was conducted, thus capturing all the tweets that were still on the platform between the above 2 dates. We then searched this data set using a Python script (Python Software Foundation) [16] to identify tweets that contain references to the terms “fact check*,” “misinformation,” “disinformation,” “fake news,” “false news,” “fakenews,” and “infodemic.” This resulted in 10,057 tweets with the above terms and in relation to vaping. These tweets were sent by 2925 unique users. After removing retweets as duplicates, 4224 tweets remained. Finally, we limited the analysis to English language tweets. The remaining tweets (n=2945) received 15,085 likes, 1075 replies, and 7761 retweets from Twitter users. These metrics are discussed in the *Results* section as indicators of how audiences engage with the content of identified tweets. Audience engagement offers an important metric on the degree of public popularity of certain topics or actors, especially in relation to different positions on vaping. It also provides an indication of the presence and possible size of online communities holding particular views on vaping.

For the manual coding of the identified tweets, 3 coders designed a codebook by using emergent coding in content analysis to a sample of the data. This process identified common categories present in the tweets [17]. Once the coders established identified recurring themes, they began to group topics into overarching ideas. It is important to mention here that the public discussion around vaping mixes the mode of delivery with its nicotine substance; accordingly, this is how we viewed the issue of vaping when classifying the tweets below.

Because of the frequency of blaming certain people or parties for spreading misinformation, the coders decided to code (1) the overall position of the tweet (provape or antivape), (2) the topics addressed, and (3) the actors blamed for spreading misinformation if applicable. Eventually, the coders refined the codebook to guide the identification of positioning on vaping using 6 exhaustive and mutually exclusive topics and 7 actors. The topics were (1) vaping is safer and more useful than alternative methods of ingesting nicotine and other psychotropic substances, and thus less harmful, for example, than cigarettes, or that it is a cessation tool; (2) vaping is as harmful as smoking and thus no safer than other tobacco products; (3) economic issues related to vaping, which included discussions of businesses closing due to new vaping restrictions and speculation of possible profit losses for tobacco companies from competing vaping companies; (4) policy action or regulation of vaping such as flavor bans, retail access, and implications of unregulated products; (5) advertising, promotion, and sponsorship of vaping products and businesses; and (6) other topics such as body autonomy, vaping in relation to the COVID-19 pandemic, and impact on minors.

The coders identified that the following actors were blamed for potentially spreading misinformation: (1) public health authorities (PHA), which included the American Health Association, World Health Organization (WHO), and individuals associated with such organizations; (2) news media, including specific news outlets, scientific publications, and authors associated with these publications; (3) government actors including government agencies, political parties, and figures, stated in specific and broad terms such as the US Department of Health and Human Services, and “the (global) government”; (4) advocacy groups supporting or opposing vaping including Tobacco Free Kids, Action on Smoking and Health, and American Vaping Association; (5) public health experts including academic researchers, medical practitioners, and officials associated with public health bodies; (6) industry actors including tobacco companies (often referred to in tweets as “Big Tobacco”) and the vaping industry; and (7) other actors including lawyers and insurance companies. Though limited, the 7 actors include the vaping industry and lobbies, which will be discussed below. The topics and actors were exhaustive and mutually exclusive. Individual tweets could be coded as containing more than 1 topic and actor.

Following the analytical steps described above, 2 coders coded a random sample of tweets (n=430) independently to test intercoder reliability. The initial coding for the above sample was statistically low for both topics and actors, and additional clarification was added to the codebook. Both coders came to an agreement that taking hashtags into consideration when coding topics was necessary to understand the subject and positioning of a tweet. This aligns with the social media research that finds the use of hashtags, on social media sites such as Twitter, serves to start web-based discourses pertaining to a subject and convey emotions, context, and associated thoughts on a particular subject [5,7,18]. As such, the coders categorized frequently used hashtags into their respective topics. For example, hashtags such as #vapingsaveslives and #hardreduction were categorized into topic 1; #MSAbloodmoney into topic 3; and #Flavorban and #vaperallydc2019 into topic 4. Because of the extensive use of hashtags, with tweets often using multiple hashtags, statistical intercoder agreement increased. Any discrepancies were discussed by the coders and reconciled. The initial low agreement was mainly due to the tagging feature available on Twitter, which allows you to mention multiple users or pages using the @ symbol to engage with them. Coders agreed that a tweet must explicitly accuse an individual or organization of spreading misinformation to be coded as such, to avoid assuming why additional users were being mentioned in a tweet. For example, the tweet “Ok. #vaping #misinformation #condescension @UnbreakableHate @EdwardHubert4 @Agent4MassGov @ChaunceyGardner...” tags 4 other users, but it is unclear if the original poster mentioned these users because they wanted to draw their attention to the tweet or because the user is implying that they are also spreading misinformation. Therefore, this tweet would not be coded as containing misinformation actors. On the other hand, the following tweet makes an explicit misinformation accusation against both the Food and Drug Administration (FDA) and news media and, therefore, was coded as containing misinformation actors (1) PHA and (2) news media:

Take note, @realDonaldTrump: These are TRUE statistics the @US_FDA doesn’t want you to know. The #FakeNewsMedia will not tell you how wonderful the #vape industry is. They listen to the agenda-driven FDA and spew misinformation.

After this adjustment to the codebook, the statistical agreement between the 2 coders increased. The intercoder reliability using Krippendorff α scores are as follows: α≥.824 for the tweets’ position from the first coding attempt, α≥.973 for all actors, and α≥.970 for all topics, both from the second coding attempt. Once these clarifications were made and the codebook was finalized, the 2516 tweets were coded by the same 2 coders. Finally, we used QDA Miner—WordStat9 (Provalis Research), which is a digital method, to further analyze some tweets. This digital tool is useful in extracting meaningful insight from unstructured data and it has been used in numerous previous research studies involving various interdisciplinary fields [19]. QDA miner—Wordstat9 can identify the most frequently used words, phrases, and their co-occurrences in Twitter posts. This computer-assisted program analyzes how words and phrases correlate with others, which is important when analyzing the meaning of qualitative data in the given contexts. Furthermore, it is useful in identifying dominant textual patterns and has been found to be both largely efficient and accurate when dealing with large amounts of data [20]. For example, salient topics can be recognized through factor analysis by ranking them using the resulting eigenvalue from the mathematical linear equation. The higher the eigenvalue is the more dominant a topic is [14].

### Ethical Considerations

Given that this study is a content analysis based on nonreactive research with data extracted from public social media posts, no ethics clearance was needed from our university. This is similar to a previous study conducted by one of the coauthors in this paper [16]. Also, our study does not specifically concern vulnerable or at-risk groups, but regardless, the Twitter user’s information, such as usernames, has not been included in our study in order to deidentify Twitter users. Also, the study does not contain any Twitter hyperlinks that can be used to identify specific users or individual tweets.

## Results

To answer the study’s research question, we found that 98.9% (2625/2653) of the tweets analyzed expressed provape positions compared to 1.1% (28/2654) expressing antivaping positions (see Table 1). Overall, 99.6% (n=21,778/21,860) of the audiences associated with these tweets engaged with provape content compared to 0.4% (82/21,860) engaging with antivape content. The average engagement for provaping tweets was 8.3 and for antivaping tweets was fewer than 3.

**Table 1:** Content analysis of the 2945 tweets referencing misinformation and audience engagement with each category^a^.

Category			Frequency, n (%)		Likes, n (%)		Replies, n (%)		Retweets, n (%)	Total engagement, n (%)		Average engagement, n
**Position** **(frequency: n=2653; likes: n=13,844; replies: n=929; retweets: n=7087; total engagement: n=21,860)**												
	Provape	2625 (98.9)		13,802 (99.6)		917 (98.7)		7059 (99.6)		21,778 (99.6)	8.3	
	Antivape	28 (1.1)		42 (0.4)		12 (1.3)		28 (0.4)		82 (0.4)	2.93	
**Topics** **(frequency: n=2022; likes: n=11,047; replies: n=679; retweets: n=5849; total engagement: n=17,575)**												
	Vaping is safe	1154 (57)		6590 (59.6)		381 (56.1)		3440 (58.8)		10,411 (59.2)	9.02	
	Vaping is harmful	3 (0.1)		24 (0.2)		3 (0.4)		16 (0.2)		43 (0.2)	14.33	
	Economic impacts	140 (6.9)		828 (7.4)		48 (7)		471 (8)		1347 (7.6)	9.62	
	Call for action	296 (14.6)		1077 (9.7)		78 (11.4)		586 (10)		1741 (9.9)	5.88	
	Advertising	56 (2.7)		425 (3.8)		29 (4.2)		248 (4.2)		702 (3.9)	12.5	
	Other	373 (18.4)		2103 (19)		140 (20.6)		1088 (18.6)		3331 (18.9)	8.93	
**Actors being blamed for spreading misinformation** **(frequency: n=1476; likes: n=7383; replies: n=521; retweets: n=3849; total engagement: n=11,753)**												
	Public health authorities	390 (26.4)		2333 (31.6)		156 (29.9)		1167 (30.3)		3656 (31.1)	9.37	
	News media	446 (30.2)		1913 (25.9)		111 (21.3)		1039 (27)		3063 (26.1)	6.87	
	Government actors	165 (11.2)		796 (10.8)		54 (10.4)		440 (11.4)		1290 (11)	7.82	
	Advocacy groups	144 (9.8)		749 (10.1)		36 (6.9)		361 (9.4)		1146 (9.8)	7.96	
	Health experts	102 (6.9)		679 (9.2)		53 (10.2)		341 (8.9)		1073 (9.1)	10.52	
	Big tobacco	54 (3.7)		228 (3.1)		35 (6.7)		104 (2.7)		367 (3.1)	6.80	
	Other	175 (11.9)		685 (9.3)		76 (14.6)		397 (10.3)		1158 (9.9)	6.62	

^a^Data were collected from Twitter between August 1, 2006 and August 12, 2022.

Among the most common topics discussed, the first claims were about vaping being safe (1154/2022, 57%), followed by other topics such as body autonomy and the COVID-19 pandemic (373/2022, 18.4%), calls for policy action (296/2022, 14.6%), and economic issues related to vaping (140/2022, 6.9%). The lowest proportion of tweets (3/2022, 0.1%) were related to the harms associated with vaping.

Total audience engagements with tweets (measured by the total number of likes, retweets, and replies for all tweets) followed the same order as the frequency cited above, with vaping is safe accounting for 59.2% (10,441/17,575) of total engagements, followed by other topics (3331/17,575, 18.9%), and calls for action (1158/17,575, 9.9%). The topic that receives the lowest proportion of total engagements (43/17,575, 0.2%) is vaping as harmful.

When analyzing engagement per tweet, vaping is harmful averaged to 14.3 replies, likes, and retweets per tweet. While vaping is safe was the topic that was the most frequently engaged with overall, it received the third lowest average engagement per tweet (9.02). The topic of advertising, promotion, and sponsorship of vaping products and businesses received the second lowest total engagements but had the second highest engagement per tweet (12.5), followed by economic impacts (9.62), other topics (8.93), and calls for policy action (5.88; see Table 1).

The actors receiving the highest total engagement were PHA (3656/11,753, 31.1%), news media (3063/11,753, 26.1%), government actors (1290/11,753, 11%), other actors (1158/11,753, 9.9%), advocacy groups (1146/11,753, 9.8%), and public health experts (1073/11,753, 9.1%). The actor with the lowest proportion of total engagements was the tobacco industry (367/11,753, 3.1%). By average engagement per tweet, however, tweets related to public health experts received an average of 10.52 replies, retweets, and likes per message, followed by PHA (9.37). Advocacy and government actors averaged 7.9 engagements, and news media, industry actors, and other actors attracted between 6.5 and 6.9 engagements on average per tweet.

On actors accused of misinformation in provaping tweets, news media are most frequently cited (446/1476, 30.2%). CNN is the most frequently mentioned (n=43), followed by Bloomberg (n=17), ABC (n=13), New York Times (n=9), and MSNBC (n=7). An example of provaping accusation of news media industry is “CNN tweet spreads vaping fallacies to millions #CNN #fakenews #Vape #vapecommunity...”

Antivaping tweets are far fewer and accuse different news outlets, notably conservative media outlets like The Sun in the United Kingdom and Fox News in the United States. For example:

@TheSun #Vaping is WORSE than #SMOKING// please go learn the facts before you print #FakeNews stories @TheSun.

As part of the “other topics” category, 1 antivaping tweet argues that social media itself is to be blamed for misinformation about vaping:

Misinformation is rampant in social media. Non qualifying people spreading “fake facts” like a virus. But you are right, it is hard to counter lies and beliefs with facts. #Vaping is not safe.

Following the news media, the actors most accused of misinformation include PHA (390/1476, 26.4%) and then government figures (165/1476, 11.2%). Most are accused of benefiting from spreading misinformation about the vaping industry. Actors accused of misinformation by antivaping tweets are the vaping industry (144/1476, 9.8%), health experts not associated with recognized PHA (102/1476, 6.9%), and the tobacco industry (54/1476, 3.7%). Analyzing tweets accusing news media actors of misinformation, 419 tweets were with provaping positioning and 3 were from the antivaping positioning.

The topic of economic issues is exclusively discussed in relation to provaping messages, for there are no tweets discussing the economic benefits of abandoning vaping. Additionally, while we found no discussion of the economic benefit to vaping as such, there was dialogue surrounding the economic disadvantages of a vape ban including the loss of tobacco taxes and the use of the hashtag #MSAbloodmoney, which coincided with the alleged motivations for spreading misinformation. Provaping tweets suggest that the spread of misinformation is causing small vaping businesses to close and jobs to be lost. There are also allegations that government actors and political interests are spreading misinformation to prolong proceeds from tobacco companies. For example, in this tweet, the hashtag #msabloodmoney is used:

#NeverForget The Lies &...; Propaganda spread by @realDonaldTrump, Our #Government...; #FakeNewsMedia to Destroy #Vaping a rival business of #BigTobacco all for #msabloodmoney ! #VapeBan #vapingsaveslives #flavorssavelives #flavorban #VapersUnite #WeVapeWeVote...

Master Settlement Agreement (MSA) refers to the 1998 agreement between the US Attorneys General and the tobacco industry, which required tobacco companies to pay billions of US dollars in liability for health care costs incurred by US state governments to treat tobacco-related disease and death. The funds received were initially intended to be earmarked for tobacco control including public education, cessation, and treatment programs. However, since 1998, these funds have been used in many states for other public services [21]. Provaping accusers of misinformation claim the MSA has become a source of general public revenue rather than a means of supporting smokers including harm reduction through the increased use of vaping products.

Other discussions surrounding the economic impacts of vaping, or rather, banning vaping referenced small business closures. For example, some provape users mentioned the following tweet:

Forbes: While Media Outlets Continue To Spread Misinformation About Electronic Cigarettes, the White house reconsiders flavor ban and the 10,000 small businesses it will affect. #prosmoke #jobs #vaping #health #smoking #tobacco #quitsmoking...

Regarding calls for policy action, there were only 3 tweets by the antivape users who demanded some action such as the following example:

#Vaping is not safe, it has serious health consequences! @Ohpediatricians created a powerful resource for teens and parents for a quick fact check on the risks of vaping and how to quit today. #vapingban...

Other antivaping discussions within this topic included a call for action to stop promoting vaping as safe or prevent marketing it to children by enticing them with vaping flavors. On the other hand, discussion from the provaping community on this topic referenced campaigns and rallies against the banning of vaping or its flavors with the use of the hashtag #flavoursavelives. Another call for action focuses on introducing better regulations on vaping products. Some tweets in this category express fear of the emergence of unsafe vaping products being sold on the black market, if vaping is not regulated properly. For example, we find the following tweet:

I love our President @charliekirk11 but his ban on flavors for #vaping is dead wrong and he’s getting the statistics from #FAKENEWS Millions of fake black market vendors will pop up causing an epidemic of illnesses if this goes through! @realDonaldTrump

On tweets concerning the topic of advertising, promotion, and sponsorship of vaping products and businesses, provaping tweets often encourage users to ignore alleged misinformation about vaping while promoting e-cigarette products. Many posts offer warnings that antivaping advocates spread misinformation on social media. Tweets on this topic include information sessions and campaigns to counter alleged misinformation on the harms of vaping. For instance, 1 tweet states:

New campaign aims to counter the misinformation around #vaping to ‘encourage more smokers to switch on to vaping.’ Read more about the campaign here [...] #nzpol #health #vaping #ecigs.

Some appropriate the term “fake news” for marketing purposes, accusing competing suppliers of vaping products of falsely offering lower prices. For example, 1 tweet states:

If you hear someone else has lower prices than us, that’s #FakeNews—We will match any lower deal you find! #vape #vaping #vapefam...

A relatively small number of provaping tweets accuse the tobacco industry of misinformation to protect its markets. Antivaping tweets referenced the vaping industry 3 times. For example:

18-year-old #CollegeStudent’s Lung COLLAPSED after #Vaping for a Year [...] #Juul #parents #BackToSchool2019 #healthcare #vapingsaveslives = #FakeNews.

Finally, our analysis finds efforts by provaping tweets to leverage political influence through voting as a potential means to pressure politicians. Accusations directed at news media are also often accompanied by expressions like #vapingsaveslives (n=60) or #wevapewevote (n=26), suggesting that vapers should exert their political voice during elections.

## Discussion

Previous studies of the spread of misinformation about vaping on social media have mostly focused on the volume of misinformation being circulated, the claims made about vaping and its accuracy, and the consequences of misinformation for vaping behaviors. While there has been growing research on the strategic use of misinformation accusations in other policy domains, this has not yet been applied to vaping. This paper conducts the first analysis of how claims about the use of misinformation and actors accused of spreading such claims have been used to support pro- and antivaping narratives.

Our findings show that provaping is the dominant positionality on Twitter when discussing misinformation surrounding the topic. Provaping tweets are more frequent and have more user engagement overall than antivaping tweets. One exception to this is the topic of vaping being harmful, which despite its scarcity, has the most engagement per tweet out of all the identified topics. On the other hand, the most dominant topic overall is that surrounding the idea of vaping being safe. Other topics around vaping include body autonomy, calls for action, and the economic impacts of vaping, specifically discourse surrounding suspicion about the beneficiaries of spreading misinformation. Both positionalities proposed news media as disseminators of misinformation although the specific outlets differ depending on the stance, PHA, and government actors. The prevalence of accusations from provape advocates surrounding the spread of misinformation about vaping on Twitter strongly shapes what is deemed to be misinformation, which specific actors are accused of spreading, and the alleged motives for doing so. Patterns of tweeting and engagement through likes, comments, and retweets further amplify these misinformation accusations.

The strategic use of misinformation accusations by provaping advocates to silence opponents is likely to have short-term benefits. Some tweets seem to have clear for-profit intent, using accusations to gain commercial advantage and increase sales. Those seeking clear policies and regulation of vaping products that support access for harm reduction purposes, while acknowledging the need to protect uptake by nonusers including youth, would benefit from the informed public debate about the pros and cons of ENDS. The evolving science on the longer-term risks and perceived benefits of vaping will continue to provide the basis for such debates. Accusations of misinformation, extending to proven sources of independent evidence such as public health researchers, can undermine informed public debate that would benefit both provaping and antivaping positions.

These findings raise important implications for protecting and promoting public health and for strategies for countering misinformation as harmful to public discourse. First, there are both risks and benefits from vaping depending on a user’s previous and current use of combustible or other tobacco products. While the uptake of vaping by nonusers of tobacco products brings harm to health, vaping to reduce or stop the use of combustible tobacco products brings health benefits. Dual use of both vaping and combustible tobacco products can prolong the latter and even increase risks. Products with high levels of nicotine can result in addiction by new users with uncertain long-term health risks. Secondhand vapor can increase health risks for those exposed unless vaping leads to a reduction in exposure to secondhand smoke. These different contributions to risk and benefit require careful navigation, informed by evolving scientific evidence, to determine the most beneficial pathways for prospective users. However, the proliferation of misinformation about vaping has created challenges for those seeking to understand these complexities by distorting available evidence. Moreover, the findings from this research suggest that, in addition to the actual spread of misinformation, accusations of the spread of misinformation by both supporters and advocates of vaping (and especially the latter) for strategic reasons, further undermine the capacity for informed public discourse. This is because the evidence from this study suggests such accusations have more to do with disagreement with opposing views than the spread of misinformation per se. Our analysis suggests that the result is a polarizing of positions on vaping in ways that prevent meaningful engagement with evidence on the balance of risks and benefits involved. Misinformation accusations, in short, blur what is deemed accurate versus false information, and who is perceived as credible and noncredible sources of information. This, in turn, hinders the informed use or nonuse of vaping products to protect and promote public health.

Furthermore, this analysis raises concerns about the broader consequences of misinformation accusations for public trust. The increased volumes of misinformation in the public domain are a well-documented problem in the early 20th century. There is also evidence of a growing proliferation of accusations of misinformation for political or economic gain. COVID-19 has seen a surge in the accusations against scientists, public health officials, government actors, political leaders, and news media. There is growing concern, however, that the weaponization of misinformation claims, with a widening breadth and frequency of such accusations, is profoundly undermining trust in public institutions [15]. This, in turn, is contributing to troubling patterns of social exclusion, marginalization, isolation and alienation which weaken the functioning of social institutions.

Given the above findings, it is imperative that we seek potential strategies to mitigate the challenges of dealing with misinformation accusations. While it may not be possible to remove all the misinformation regarding vaping on Twitter, efforts could be made to filter information and develop media literacy. On the first point, 1 study on misinformation about COVID-19 identified a successful approach to combat misinformation via reducing the visibility of certain content or adding warning labels to content that possibly contained inaccurate or harmful information [22]. On the second point, users’ media literacy skills can be enhanced by equipping them with the necessary tools to fact-check information and encourage them to reflect upon their own experiences and beliefs before disseminating misinformation. Platforms and policy makers should provide users with more resources for verifying web-based content as increased media literacy would lead to more social media dissemination of accurate information [23].

While this paper has begun to interrogate the nature of misinformation accusations related to vaping on Twitter, there are some limitations to address. Our analysis is based on tweets that contain the hashtags #vape and #vaping as well as mentions of misinformation and other fake news-related terms; however, there may be additional tweets discussing vaping misinformation or disseminating misinformation that uses the terms vape or vaping without these hashtags. More so, we did not analyze the other types of content referenced in some tweets such as web-based articles and editorials, for there is a possibility that these additional types of content may give further insight into the tone (ie, sarcasm) and the lack of context may lead to misunderstanding some tweets. Also, our study is limited to Twitter, and social media discourse may differ across other platforms. Finally, there may be a limitation to our data collection time line. Tweets were collected up until August 12, 2022, and web-based discourse may slightly differ if more recent data are taken into consideration.

There is a need for further research to analyze other social media platforms such as TikTok and Instagram that are popular platforms for vaping-related content. This paper’s analysis of English-language tweets can be expanded to other languages relevant to jurisdictions where public discourse about vaping use is relevant. Cross-national comparisons of misinformation accusations are needed to understand whether this is a largely US-based phenomenon or a global concern. Importantly, the findings of this research indicate the need for strategies to, not only counter the spread of misinformation but also counter false accusations of misinformation.

## Abbreviations

API: application programming interface
ENDS: electronic nicotine delivery systems
FDA: Food and Drug Administration
MSA: Master Settlement Agreement
PHA: public health authorities
WHO: World Health Organization

## Data Availability

The data sets generated and analyzed during this study are available from the corresponding author on reasonable request.
